# Supplementary material for: Electrophysiological Responses from the Human Tongue to the Six Taste Qualities and Their Relationships with PROP Taster Status
Source: Nutrients. 2020 Jul 7;12(7):2017. doi: 10.3390/nu12072017 (PMC7400817; doi:10.3390/nu12072017)
Supplement: Supplementary file 1 [file nutrients-12-02017-s001.pdf]

Supplementary Material:

Table S1. Ratings of perceived taste intensity in response to three concentrations of PROP and NaCl in the taster groups.

	**Super-taster (n = 10)**	**Medium taster (n = 15)**	**Non-tasters (n = 14)**
**PROP (mmol/L)**			
0.032	11.99 ± 2.74	4.58 ± 1.26	0.97 ± 0.46
0.32	47.39 ± 4.69*	36.23 ± 3.94	3.73 ± 0.94*
3.2	86.50 ± 3.80*	64.83 ± 4.98	25.66 ± 4.71*
**NaCl (mol/L)**			
0.01	1.44 ± 0.43	2.36 ± 1.33	3.30 ± 1.23
0.1	26.50 ± 5.69*	31.59 ± 3.52	31.13 ± 4.33*
1	57.75 ± 6.45*	71.86 ± 4.73	60.14 ± 4.03*

Values are means ± SEM. n = 39. Three-way ANOVA was used to compare PROP intensity ratings with NaCl intensity ratings across groups ($F_{4,217}$= 6.248; p = 0.00009).
* = significant difference between PROP and the corresponding NaCl concentration (p < 0.00017; Duncan's test).
